# Genetic Diversity of Microneme Protein 2 and Surface Antigen 1 of *Eimeria tenella*

**DOI:** 10.3390/genes12091418

**Published:** 2021-09-15

**Authors:** Tuấn Cường Võ, Haung Naw, Rochelle A. Flores, Hương Giang Lê, Jung-Mi Kang, Won Gi Yoo, Woo-Hyun Kim, Wongi Min, Byoung-Kuk Na

**Affiliations:** 1 Department of Parasitology and Tropical Medicine, Institute of Health Sciences, Gyeongsang National University College of Medicine, Jinju 52727, Korea; vtcuong241@gmail.com (T.C.V.); haungnaw23@gmail.com (H.N.); gianglee291994@gmail.com (H.G.L.); gjm9951001@hanmail.net (J.-M.K.); wgyoo@gnu.ac.kr (W.G.Y.); 2 Department of Convergence Medical Science, Gyeongsang National University, Jinju 52727, Korea; 3 Institute of Animal Medicine, College of Veterinary Medicine, Gyeongsang National University, Jinju 52828, Korea; floresrochellea@gmail.com (R.A.F.); woohyun.kim@gnu.ac.kr (W.-H.K.)

**Keywords:** coccidiosis, *Eimeria tenella*, microneme protein 2, surface antigen 1, genetic diversity, natural selection

## Abstract

Avian coccidiosis is a disease caused by members of the genus *Eimeria*. Huge economic losses incurred by the global poultry industry due to coccidiosis have increased the need for cost-effective and easily available recombinant vaccines. Microneme protein 2 (MIC2) and surface antigen 1 (SAG1) of *E. tenella* have been recognised as potential vaccine candidates. However, the genetic diversity of the antigens in field isolates, which affects vaccine efficacy, has yet to be largely investigated. Here, we analysed genetic diversity and natural selection of *etmic2* and *etsag1* in Korean *E. tenella* isolates. Both genes exhibited low levels of genetic diversity in Korean isolates. However, the two genes showed different patterns of nucleotide diversity and amino acid polymorphism involving the *E. tenella* isolates obtained from different countries including China and India. These results underscore the need to investigate the genetic diversity of the vaccine candidate antigens and warrant monitoring of genetic heterogeneity and evolutionary aspects of the genes in larger numbers of *E. tenella* field isolates from different geographical areas to design effective coccidial vaccines.

## 1. Introduction

Avian coccidiosis is an enteric disease caused by infections due to *Eimeria* spp. The annual loss of the global poultry industry due to coccidiosis has been estimated at GBP 10.36 billion based on total production losses and approximate costs of prophylaxis and treatment [1]. *Eimeria* infection has also been known to exacerbate the infections associated with other pathogens such as *Clostridium perfringens*, resulting in severe necrotic enteritis [2]. Seven different species of *Eimeria*, namely, *E. acervulina*, *E. tenella*, *E. maxima*, *E. mitis*, *E. praecox*, *E. brunetti*, and *E. neacatrix*, are known to infect domestic chicken and cause avian coccidiosis, with varying levels of fecundity, pathogenicity, and sites of replication within the chicken gut [3]. *E. tenella* is one of the most virulent species and is economically important worldwide [4,5,6]. Anticoccidial compounds such as ionophores have been widely used in chemoprophylaxis to control *Eimeria* infection [7,8]. However, the emergence and spread of drug-resistant strains have threatened the sustainable development of the poultry industry [9]. Further, the residual anticoccidial drugs in poultry products such as meat and eggs have also been concerned as potential risks that may affect human health. In this respect, the development of effective and economic coccidial vaccines is imperative. Live attenuated coccidial vaccines have been widely utilised for almost 70 years, but are a relatively expensive cost and are difficult to scale up [10]. Recombinant vaccines are inexpensive and easily accessible and thus overcome these limitations by eliminating undesirable contaminants [11]. Therefore, several attempts have been made to develop recombinant coccidial vaccines; however, commercially available recombinant vaccine has yet to be developed.

Microneme protein 2 (MIC2) and surface antigen 1 (SAG1) of *Eimeria* are potential vaccine candidates against coccidiosis. Micronemes are secretory organelles located at the apical end of apicomplexan protozoans including *Plasmodium* spp., *Toxoplasma gondii*, *Cryptosporidium* spp., and *Eimeria* spp. [12]. MIC2 belongs to a family of microneme proteins, which play a crucial role in host cell identification, binding, and invasion of the parasite [13,14,15]. MIC2 of *E. tenella* (EtMIC2) is immunogenic and elicits protective immunity in challenge experiments, suggesting a promising coccidial vaccine candidate [16,17,18,19,20]. Surface antigens (SAGs) are a family of developmentally regulated glycosyl-phosphatidylinositol (GPI)-linked proteins in *Eimeria* [21]. Although the precise biological functions of SAGs remain unclear, they are likely to play a critical role in host–parasite interaction via attachment of the parasite and its invasion to host cells [21]. EtSAG1 is an immunodominant sporozoite antigen of *E. tenella* and is closely involved in the initiation of the infection [22].

Although EtMIC2 and EtSAG1 are prospective vaccine candidates, it is also essential to determine the genetic variation of the antigens in field isolates to design an efficient and protective vaccine since genetic variation may influence the effectiveness of vaccines. However, molecular genetic approaches to analyse the genetic diversity of the two vaccine candidate antigens have been largely neglected and only limited information of the genetic make-up of the antigens in *E. tenella* field isolates is available. In this study, we analysed the genetic diversity of *etmic2* and *etsag1* in *E. tenella* Korean isolates to delineate the genetic heterogeneity of the two genes encoding the plausible vaccine candidate antigens.

## 2. Materials and Methods

### 2.1. Chicken Faeces Samples and Genomic DNA Extraction

A total of 367 chicken faeces samples were collected from the poultry farms of Korea in 2020–2021. Most samples were collected from broilers (75.8%) and the remaining were from layers (11.9%) or breeders (12.3%). All the chickens were not vaccinated with any kind of coccidial vaccine. The faeces were screened microscopically to detect *Eimeria* infections. The faecal samples that harboured more than 100 oocysts per gram were selected for further analysis. The oocysts of the parasites were collected from the faecal samples via saturated sodium chloride flotation and sporulation method [4]. Genomic DNA was extracted from the collected oocysts as follow. To destruct oocyst wall, the oocysts were suspended in 500 µL lysis buffer (10 mM Tris-HCl, pH 8.0, 25 mM ethylenediaminetetraacetic acid (EDTA), 100 mM NaCl, 0.5% sodium dodecyl sulfate (SDS), and 0.5 mg/mL proteinase K). The oocysts were ground by vortexing with Ø 0.5 mm glass bead (BioSpec Product Inc., Bartlesville, OK, USA) for 10 min. The supernatant was transferred to a new tube containing 500 µL ASL buffer (Qiagen, Hilden, Germany) and incubated at 56 °C overnight. Genomic DNA was purified from the sample using QIAamp Fast DNA Stool Mini Kit (Qiagen, Hilden, Germany) following the manufacturer’s protocols. Species-specific nested polymerase chain reaction (PCR) targeting *ITS-1* was performed to identify the *Eimeria* species [23]. The faecal samples harbouring *E. tenella* were finally selected and used for further study.

### 2.2. Amplifications of etmic2 and etsag1

Amplifications of *etmic2* and *etsag1* were performed via nested PCR from the genomic DNA of *E. tenella*-positive samples. The primers and thermal cycling conditions of the amplification are listed in Table S1. The *etmic2* primers flanked almost full-length genes, in which 81 nucleotides encoding 27 amino acids including the N-terminal signal peptide region were depleted. The primers for *etsag1* were designed to amplify the complete ectodomain of EtSAG1 corresponding to amino acids 24 to 235. Ex *Taq* DNA polymerase (Takara, Otsu, Japan) with proof-reading activity was used in all PCR steps to minimise the nucleotide mismatching during the amplification. Each PCR product was analysed via 1.5% agarose gel electrophoresis, purified from the gel, and cloned into a T&A cloning vector (Real Biotech Corporation, Banqiao City, Taiwan). Each ligation mixture was transformed into *Escherichia coli* DH5α competent cells and positive clones with appropriate insert were selected via colony PCR with nested PCR primers. The nucleotide sequences of the cloned *etmic2* and *etsag1* were analysed using the automatic Sanger method with M13 forward and reverse primers. Plasmids from at least two independent clones from each isolate were analysed to confirm the sequence accuracy. Introns in each gene sequence were manually deleted based on the reference sequences, *E. tenella* Houghton strain *etmic2* (XM_013377912.1) and *etsag1* (AJ586531.2). The nucleotide sequences analysed in this study were deposited in GenBank under the accession numbers MZ576583–MZ576738 for *etmic2* and MZ576739–MZ576839 for *etsag1*.

### 2.3. Analyses of Genetic Diversity and Natural Selection in Korean etmic2 and etsag1

The nucleotide and deduced amino acid sequences of Korean *etmic2* and *etsag1* were analysed using Editseq and Seqman programs in the DNASTAR package (DNASTAR, Madison, WI, USA). The number of segregating sites (S), haplotypes (H), haplotype diversity (Hd), nucleotide diversity (π), and the average number of pair-wise nucleotide differences within a population (*K*) were calculated with DnaSP ver. 5.10.00 [24]. The rates of synonymous (dS) and nonsynonymous (dN) substitutions were calculated and compared using the Z-test (*p* < 0.05) with MEGA6 program [25] based on Nei and Gojobori’s method [26] with the Juke and Cantor (JC) correction of 1000 bootstrap replications. Tajima’s D test [27] and Fu and Li’s D and F statistical analyses [28] were performed using DnaSP ver. 5.10.00 [24] to evaluate the neutral theory of natural selection. The putative B-cell epitopes in EtMIC2 and EtSAG1 were predicted by the Antibody Epitope Prediction program from IEDB Analysis Resource (http://tools.iedb.org/main/bcell/; accessed on 12 May 2021) with the threshold value of 0.5 to assess the relationship between genetic diversities in the two target genes within *E. tenella* isolates and the host immune pressure [29]. Genetic variations of *etmic2* and *etsag1* in field isolates from other countries were also compared to determine the genetic diversity of the two genes in the population. The analysis included sequences available in public database; China *etmic2* (*n* = 21), India *etmic2* (*n* = 1), China *etsag1* (*n* = 21), and India *etsag1* (*n* = 2) (Table S2). To analyse the amino acid polymorphism in EtMIC2 and EtSAG1 among *E. tenella* field isolates from other geographical origins, a logo plot was constructed for each population using the WebLogo program (https://weblogo.berkeley.edu/logo.cgi; accessed on 28 April 2021). Hydrophobicity plot analysis was performed with the ProtScale program from the ExPASy server (https://web.expasy.org/protscale/; accessed on 10 May 2021) using the Kyte & Doolittle scale [30].

## 3. Results

### 3.1. Amplifications of etmic2 and etsag1 in Korean E. tenella

Species-specific nested PCR analysis of *Eimeria ITS-1* in 367 chicken faecal samples suggested that 231 were infected with *E. tenella*. Most of them were mixed infections with other *Eimeria* species including *E. acervulina* and/or *E. maxima*. Nested PCR trials to amplify *etmic2* and *etsag1* from the samples revealed that 156 *etmic2* and 101 *etsag1* fragments were successfully amplified from 231 *E. tenella*-positive samples. The approximate sizes of the amplified *etmic2* and *etsag1* were 1200 and 980 bp, respectively, and no size polymorphism was detected between and among the amplified products. Sequence analysis of each cloned gene fragment revealed accurate amplification of the target genes. The co-ding region of each gene was annotated by deleting non-coding internal introns manually based on the reference sequences, *E. tenella* Houghton strain *etmic2* (XM_013377912.1) and *etsag1* (AJ586531.2). The length of the annotated coding region of all 156 *etmic2* was 948 bp coinciding with the expected size. The size of annotated *etsag1* encoding the ectodomain region was 640 bp, and no size polymorphism was detected among and between the 101 *etsag1* fragments.

### 3.2. Polymorphism Patterns in Korean etmic2 and etsag1

**Figure 1:**
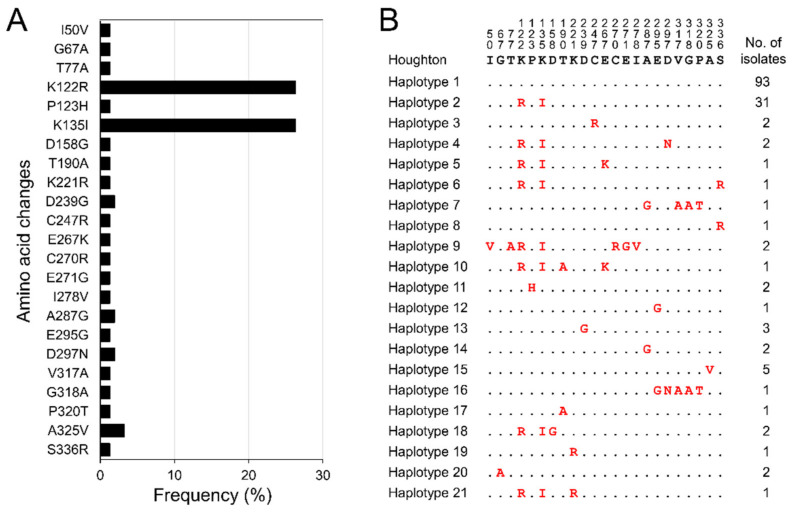
Amino acid sequence polymorphisms of *etmic2* in Korean *E. tenella* isolates. (**A**) Amino acid changes. A total of twenty-three amino acid changes were detected in Korean *etmic2*. (**B**) Haplotype diversity. Twenty-one distinct haplotypes were identified in 156 Korean *etmic2* sequences. The dots represent residues identical to the reference sequence of the Houghton strain (XM_013377912.1). Amino acid changes at particular amino acid positions are indicated as red.

**Figure 2:**
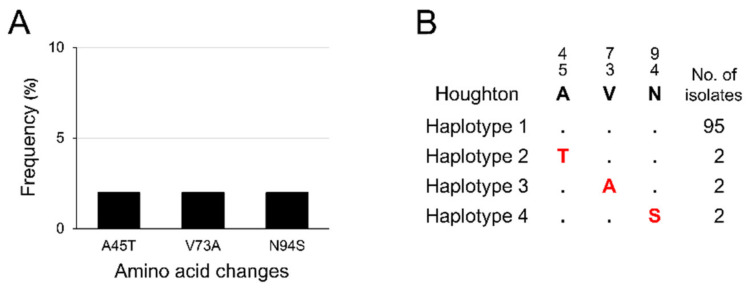
Amino acid sequence polymorphism of *etsag1* in Korean *E. tenella* isolates. (**A**) Amino acid changes. Three different amino acid changes were detected in Korean *etsag1*. (**B**) Haplotype diversity. Four distinct haplotypes were identified in 101 Korean *etsag1* sequences. The dots represent residues identical to the reference sequence of the Houghton strain (AJ586531.2). Amino acid changes at particular amino acid positions are indicated as red.

When deduced amino acid sequences of 156 Korean *etmic2* were compared with the Houghton strain reference sequence, dimorphic amino acid polymorphisms at 23 positions were identified in Korean *etmic2* (Figure 1A). These amino acid changes were distributed unevenly across the gene. K122R and K135I were the most predominant amino acid changes observed at a frequency of 26.3% (*n* = 41) in Korean *etmic2*. The other 21 amino acid changes were minor and detected only in two, three, or five sequences. Sequence alignment of 156 Korean *etmic2* classified the sequences into 21 distinct haplotypes at the amino acid level (Figure 1B). Haplotype 1, which was identical to the reference sequence of the Houghton strain, was the most predominant (*n* = 93, 59.6%). Haplotype 2–harbouring K122R and K135I was the second most prevalent haplotype in Korean *etmic2*. The other 19 haplotypes were detected at a low frequency ranging from 0.6% to 3.2%. Interestingly, two major amino acid changes (K122R and K135I) were observed simultaneously in all haplotypes carrying the variations. Meanwhile, Korean *etsag1* showed extremely limited amino acid polymorphism (Figure 2). Only three dimorphic amino acid changes with low frequencies were detected in 101 Korean *etsag1*; A45T (*n* = 2), V73A (*n* = 2), and N94S (*n* = 2) (Figure 2A). Based on amino acid sequences, Korean *etsag1* sequences were classified into four different haplotypes (Figure 2B). Most Korean *etsag1* (*n* = 95, 94.1%) sequences were identical with the reference sequence of the Houghton strain. Three amino acid changes, A45T, V73A, and N94S, were individually identified in the haplotypes 2–4, respectively.

### 3.3. Polymorphism Patterns of etmic2 and etsag1 in E. tenella with Different Geographical Origins

**Figure 3:**
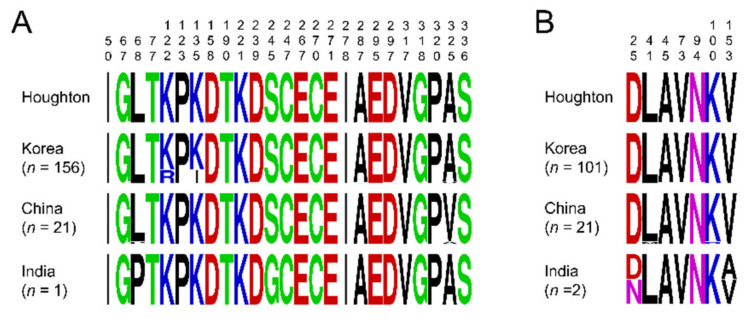
Comparative analysis of amino acid polymorphisms of *etmic2* and *etsag1* in Korean, Chinese, and Indian *E. tenella* isolates. (**A**) Amino acid polymorphisms in *etmic2*. (**B**) Amino acid polymorphisms in *etsag1*. The patterns of amino acid changes differed by country. The logo plot for each gene was constructed using the WebLogo program. The sequences of Houghton *etmic2* (XM_013377912.1) and *etsag1* (AJ586531.2) were used as reference sequences. Amino acid positions were marked on top of each amino acid change.

Database search revealed that only few sequences of *etmic2* and *etsag1* for *E. tenella* field isolates originating in other countries were currently available, including China *etmic2* (*n* = 21), India *etmic2* (*n* = 1), China *etsag1* (*n* = 21), and India *etsag1* (*n* = 2). Overall amino acid polymorphisms of *etmic2* and *etsag1* in the isolates were compared to the reference sequences of the Houghton strain and sequences of Korean isolates (Figure 3). The *etmic2* revealed low levels of amino acid polymorphism among and between the analysed sequences. Twenty-one Chinese *etmic2* sequences showed two dimorphic amino acid substitutions, L68P (*n* = 1) and A325V (*n* = 20) (Figure 3A). A325V was also detected in five Korean *etmic2* sequences. Meanwhile, the Indian *etmic2* sequence carried two dimorphic amino acid changes, L68P and S245G. The *etsag1* showed limited levels of amino acid polymorphism in the analysed sequences (Figure 3B). Only seven amino acid changes with low frequencies were found in the analysed sequences, which were not evenly distributed in the isolates from the three countries, Korea, China, and India. L41M and K100R were identified in Chinese *etsag1*, and D25N and V153A were found in Indian *etsag1*. The three amino acid changes, A45T, V73A, and N94S, identified in Korean *etsag1* at very low frequencies were not found in the sequences from China and India.

### 3.4. Nucleotide Diversity and Natural Selection of Korean etmic2 and etsag1

**Figure 4:**
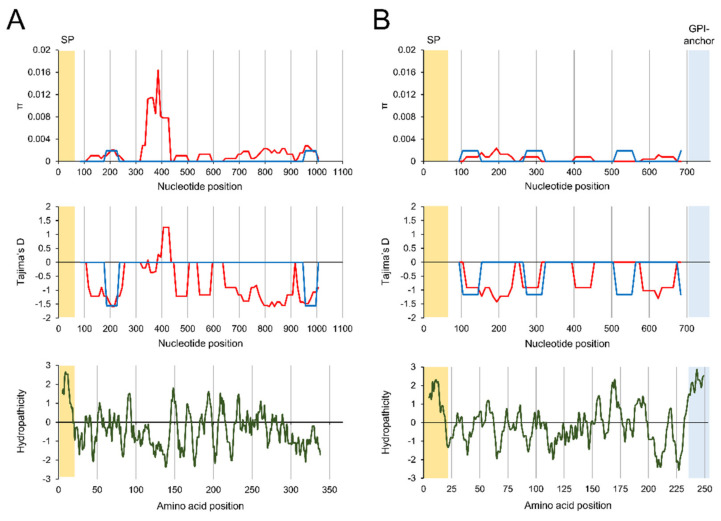
Nucleotide diversity and natural selection of *etmic2* and *etsag1*: (**A**) *etmic2*; (**B**) *etsag1*. Sliding window plot analyses for nucleotide diversity (π) and Tajima’s D across *etmic2* and *etsag1* were performed. The window size of 50 bp and a step size of 10 bp were used. Red lines, Korean *etmic2* and *etsag1*; blue lines, Chinese *etmic2* and *etsag1*. Hydropathy analysis for each protein was also performed. SP: signal peptide.

The nucleotide diversity and genetic differentiation of the two genes from Korea and China were analysed (Table 1). The average number of nucleotide differences (*K*), overall haplotype diversity (Hd), and nucleotide diversity (π) in the Korean *etmic2* were 1.76, 0.674 ± 0.041, and 0.0019 ± 0.0002, respectively. Meanwhile, these values were 0.19, 0.095 ± 0.084, and 0.0002 ± 0.0002, respectively, in Chinese *etmic2*, which suggested that the overall genetic diversity of *etmic2* was greater in Korean *E. tenella* than in Chinese *E. tenella*, but different sample sizes between the two countries need to be considered. However, the estimated dN – dS values of both populations were close to zero, suggesting that they were not affected by strong natural selection. Meanwhile, Tajima’s D values for both Korean and Chinese *etmic2* were −2.0503 (*p* < 0.05) and −1.5141 *(p* > 0.1), respectively, indicating that the genes in both populations were influenced by selective sweep, but the value was not statistically significant in Chinese *etmic2*. Nucleotide diversity and neutrality were also observed in *etsag1*. The average number of nucleotide differences (*K*), overall haplotype diversity (Hd), and nucleotide diversity (π) were 0.29, 0.258 ± 0.058, and 0.0005 ± 0.0001, respectively, in Korean *etsag1*. Chinese *etsag1* shared similar low values of the parameters with Korean *etsag1*. The dN – dS values of *etsag1* in both populations were also close to zero; however, Tajima’s D values were negative both in Korean (−1.9600, *p* < 0.05) and Chinese (−1.8733, *p* < 0.05). Sliding window analysis of π across the Korean *etmic2* showed a peak at nucleotide positions between 325 and 425 calculated from full-length sequences of *etmic2* (Figure 4A). This region also revealed a positive value of Tajima’s D, suggesting that this region was under balancing selection. The hydrophobicity plot suggested that the region corresponding to 109–141 amino acid residues had strong hydrophilicity, implying that this region may be exposed to the surface of EtMIC2. Meanwhile, Chinese *etmic2* showed different patterns of sliding window: two small peaks with positive π values at the 5’-region (nucleotide positions from 185 to 225) and 3’-region (nucleotide positions from 955 to 995) (Figure 4A). The pattern of Tajima’s D also differed from Korean *etmic2*. Two strong negative peaks, which correspond to the positions of two positive π peaks, were identified in Chinese *etmic2*. In the cases of *etsag1*, no conspicuous peak of π was detected across the gene (Figure 4B). Only low positive values of π peaks were identified both in Korean and Chinese *etsag1* and these positions were strongly correlated with negative peaks of Tajima’s D. Hydrophobicity plot revealed that these regions had positive or negative values of hydropathicity at amino acid levels, suggesting surface exposure or groove formation by folding into the internal side of EtSAG1.

### 3.5. Association between Genetic Polymorphisms and Host Immune Pressure

**Figure 5:**
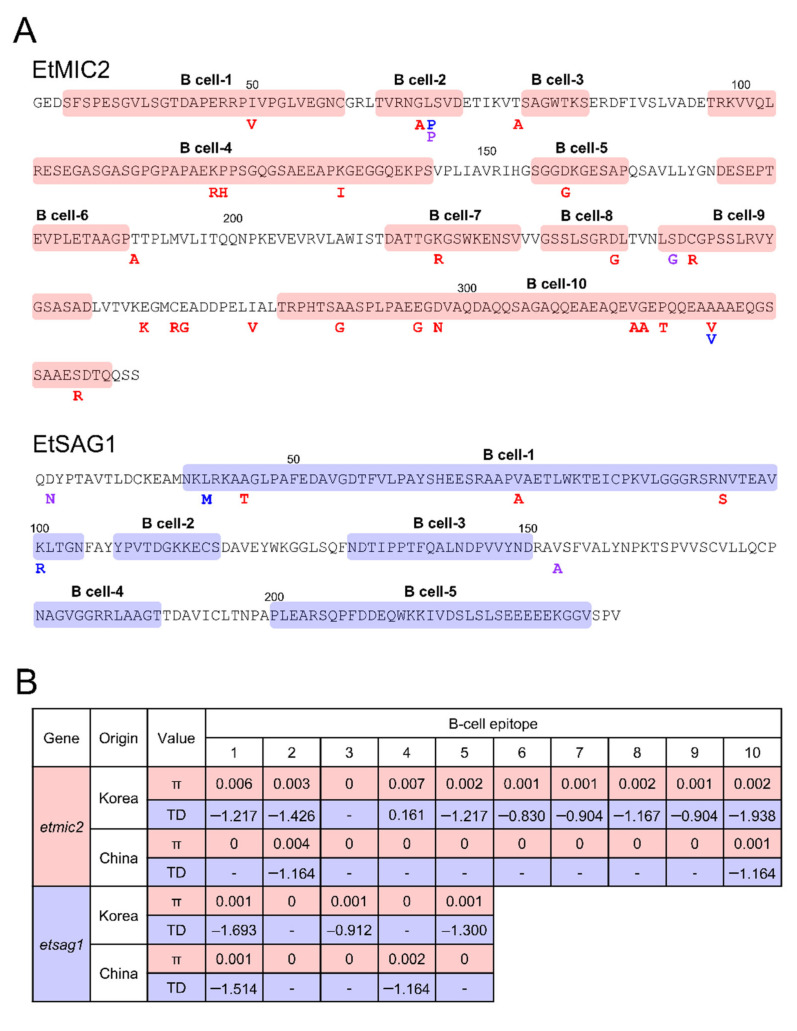
Association between genetic diversity of EtMIC2 and EtSAG1 and host immune pressure. (**A**) Positions of amino acid changes found in the putative B cell epitopes in EtMIC2 and EtSAG1. The putative B cell epitopes were boxed with pale red (EtMIC2) and pale blue (EtSAG1). Polymorphic amino acid residues detected in EtMIC2 and EtSAG1 were presented under the mutated positions with different colours (Red, Korean; Blue, Chinese; Purple, Indian). (**B**) Nucleotide diversity and natural selection analysis. Nucleotide diversity (π) and Tajima’s D (TD) values for each B-cell epitope region of EtMIC2 and EtSAG1 were analysed using the DnaSP program.

The selective pressure of host immunity on EtMIC2 and EtSAG1 was evaluated by analysing polymorphisms in the putative B-cell epitopes. A total of 10 putative B-cell epitopes were predicted in EtMIC2 (Figure 5A). Most amino acid changes identified in Korean, Chinese, and Indian *etmic2* were located in the putative B-cell epitopes. Nine of ten putative B-cell epitopes were polymorphic and showed different values of Tajima’s D. In particular, the putative B-cell epitopes 2 and 10 carrying different amino acid changes from the three countries revealed strong negative values of Tajima’s D (Figure 5B). The putative B-cell epitope 4 harbouring the major amino acid polymorphisms of Korean isolates showed a positive value of Tajima’s D, but was not statistically significant (*p* > 0.1). In the case of EtSAG1, amino acid changes identified in Korean and Chinese *etsag1* were located in putative B-cell epitope 1, which showed a strong negative value of Tajima’s D (Figure 5A,B). Meanwhile, two amino acid changes detected in Indian EtSAG1 were localised to non-B-cell epitopes.

## 4. Discussion

The rapid growth of the global poultry industry has been accompanied by huge economical losses due to coccidiosis, underscoring the need for an effective and affordable coccidial vaccine. Efforts to develop efficient and cost-effective coccidial vaccines such as recombinant and DNA vaccines have been made to overcome the drawbacks of classical live attenuated vaccines [11]. Recombinant and DNA vaccines are generally convenient to manufacture at low prices. Diverse candidate antigens have been investigated for coccidial vaccine development, raising the expectations for the development of effective vaccines [11,31]. However, natural genetic polymorphisms of vaccine candidates potentially affect vaccine efficacy [30]. Therefore, it is essential to monitor the genetic diversity of potential vaccine candidates within the global *Eimeria* population. Until now, few studies have evaluated the genetic diversity of vaccine candidate antigens in *Eimeria* field isolates. The gene encoding the immune mapped protein-1 of *E. tenella* (*etimp-1*) showed low nucleotide diversity in *E. tenella* isolates from several countries including China, India, UK, and the USA [32]. The Chinese *E. tenella* isolates exhibited limited diversity of *etmic2* and *etsag1* [33]. However, a large-scale analysis of the genetic diversity of potential vaccine candidate antigens is necessary to elucidate the genetic profile of the genes in field isolates and to facilitate the design of optimised vaccine formulations.

In this study, the genetic polymorphism and natural selection of *etmic2* and *etsag1* in Korean *E. tenella* isolates were analysed. Overall, the two genes were well conserved in Korean *E. tenella* isolates. Only limited numbers of non-synonymous nucleotide substitutions underlying amino acid changes with low frequencies were identified in both Korean *etmic2* and *etsag1*. Interestingly, two major amino acid changes, K122R and K135I, were predominantly detected in Korean *etmic2*, but not in Chinese and Indian *etmic2*. Meanwhile, the amino acid changes identified in Chinese and Indian *etmic2* and *etsag1* were not detected in Korean *etmic2*. The *etsag1* showed limited polymorphisms in Korean, Chinese, and Indian isolates than *etmic2*. Only seven amino acid changes were identified in Korean, Chinese, and Indian *etsag1*, representing country-specific changes. The overall nucleotide diversity of *etmic2* and *etsag1* was also not high in the analysed sequences from Korea, China, and India, suggesting that the two genes were relatively well conserved in the studied *E. tenella* population. Tajima’s D values for both Korean and Chinese *etmic2* were negative, suggesting that the genes were influenced by bottleneck or selective sweep in both populations. Furthermore, the dN – dS of both genes in Korean and Chinese population indicated no or minimal purifying selection. High genetic conservation of *etmic2* and *etsag1* may be due to the importance of the functional conservations of the proteins rather than escaping host immune responses to maintain essential biological functions of the proteins. Similar patterns of low levels of genetic diversity and no significant evidence of balancing selection were previously reported in the apical membrane antigen 1 of *E. tenella* [34].

Analyses of nucleotide diversity and natural selection in *etmic2* and *etsag1* also yielded several interesting findings involving the genes among the field isolates. Two amino acid changes, K122R and K135I, were identified in Korean *etmic2* with relatively high frequencies of 26.3%. The region harbouring the two amino acid changes showed a high value of π and a positive value of Tajima’s D, suggesting positive natural selection. Hydropathicity analysis suggested that the region flanking K122R and K135I may be exposed to the surface of EtMIC2, and this region strongly overlapped with the putative B-cell epitope 4. Although the precise function and structure of this region has yet to be determined, the findings suggest that it is a potential target for the host immune system. Thus, amino acid changes in this region should be carefully considered when designing an EtMIC2-based vaccine. Further, most amino acid changes identified in Korean, Chinese, and Indian *etmic2* were localised in the putative B-cell epitopes of EtMIC2, suggesting the need to consider these amino acid changes when developing coccidial vaccines based on this antigen. The *etsag1* was highly conserved among all the studied isolates. Only seven amino acid changes were identified, but five of them were localised to the putative B-cell epitope. Particularly, the Korean N94S and Chinese K100R were localised on the EtSAG1 loop, which is the target region of a sporozoite neutralizing monoclonal antibody [22]. Although the frequencies of these amino acid changes were not high in the studied population, the effect of these amino acid changes on antigen recognition and neutralisation of the sporozoites by the antibody requires further evaluation. It is also noteworthy that further evaluation of whether the putative B-cell epitopes were correctly predicted would be necessary because the B-cell epitope prediction program applied in this study is based on the B-cell epitope prediction in mammals. The different patterns of nucleotide diversity and natural selection of *etmic2* and *etsag1* in Korean, Chinese, and Indian isolates also suggested possible genetic heterogeneity of the global *E. tenella* field isolates. Further analysis of genetic heterogeneity and the evolutionary aspects of the two genes encoding potential vaccine candidates should involve larger numbers of field isolates from different geographical areas.

This study also has its limitations. Although EtMIC2 and EtSAG1 have been recognised as promising vaccine candidates for coccidiosis, only limited sequence information for *etmic2* and *etsag1* was available. Due to this limitation, we cannot establish that the two genes are highly conserved in the global *E. tenella* population, although the two genes were tightly conserved in the sequences analysed in this study. Further comprehensive molecular approaches are required to analyse the genetic diversity of the two genes in larger numbers of global *E. tenella* isolates to gain in-depth insight into their genetic profiles and facilitate the design of vaccines based on the antigens.

## 5. Conclusions

Population genetic analysis of two vaccine candidate antigens, *etmic2* and *etsag1*, demonstrated that the two genes were highly conserved in *E. tenella* field isolates. However, the two genes showed different patterns of nucleotide diversity and amino acid polymorphism in the *E. tenella* populations from different countries. Further, most amino acid changes identified in Korean, Chinese, and Indian *etmic2* and *etsag1* were mainly localised to the putative B-cell epitopes, suggesting the need to consider these amino acid changes when developing coccidial vaccines based on these antigens. The results of this study widen our knowledge of the genetic nature of the two vaccine candidates in *E. tenella* field isolates. However, studies involving larger numbers of *E. tenella* field isolates from different geographical areas are needed to determine the genetic heterogeneity and evolution of the two genes to design optimised coccidial vaccines based on these antigens.

## Figures and Tables

**Table 1:** Genetic polymorphism and tests of neutrality in the *etmic2* and *etsag1* of Korean and Chinese *E. tenella* isolates.

Gene	Country	*n*	*K*	S	Eta	H	Hd ± SD	π ± SD	dN – dS	Tajima’s D	Fu & Li’s D	Fu & Li’s F
*etmic2*	Korea	156	1.76	33	33	30	0.674 ± 0.041	0.0019 ± 0.0002	0.000	−2.0503 ^a^	1.3531 ^b^	−0.0557 ^b^
	China	21	0.19	2	2	2	0.095 ± 0.084	0.0002 ± 0.0002	0.000	−1.5141 ^b^	−2.0800 ^c^	−2.2142 ^c^
*etsag1*	Korea	101	0.29	8	8	10	0.258 ± 0.058	0.0005 ± 0.0001	−0.001	−1.9600 ^a^	0.4505 ^b^	−0.4427 ^b^
	China	21	0.38	4	4	2	0.095 ± 0.084	0.0006 ± 0.0005	−0.001	−1.8733 ^a^	−2.6730 ^a^	−2.8255 ^a^

*n*, number of analysed sequences; *K*, average number of nucleotide differences; S, number of segregating sites; Eta, total number of mutations; H, number of haplotypes; Hd, haplotype diversity; π, observed average pairwise nucleotide diversity; dN, rate of non-synonymous mutations; dS, rate of synonymous mutations; SD, standard deviation. ^a^*p* < 0.05; ^b^ *p* > 0.1; ^c^ 0.05 < *p* < 0.1.

## Data Availability

The data supporting the conclusions of this article are provided within the article. The original datasets analyzed in this study are available from the corresponding author upon request. The nucleotide sequences reported in this study have been deposited in the GenBank database under the accession numbers MZ576583–MZ576738 for *etmic2* and MZ576739–MZ576839 for *etsag1*.

## Supplementary Materials

The following are available online at https://www.mdpi.com/article/10.3390/genes12091418/s1, Table S1: List of primers used in this study, Table S2: List of sequences analysed in this study.
