# Supplementary material for: Genetic Diversity of Microneme Protein 2 and Surface Antigen 1 of Eimeria tenella
Source: Genes (Basel). 2021 Sep 15;12(9):1418. doi: 10.3390/genes12091418 (PMC8470435; doi:10.3390/genes12091418)
Supplement: Supplementary file 1 [file genes-12-01418-s001.zip › Eimeria_Vo et al._Supplementary Materials_Table S1.pdf]

**Table S1.** List of primers used in this study.

| Gene | Primer | Sequence (5'-3') | Amplicon size (bp) | Thermal cycle |
|---|---|---|---|---|
| *etmic2* | EtMIC2F | CAAGAAGTATCTTAAGCCTGC | 1291 | • 95 °C 5 min<br>• 30 cycles of (95 °C 1 min, 52 °C 1 min, 72 °C 1.5 min)<br>• 72 °C 5 min |
| | EtMIC2R | GCTTGAACCTCTTCCAGATC | | |
| | EtMIC2NF | CAGGCGAAGATAGCTTCTCTCC | 1188 | • 95 °C 5 min<br>• 35 cycles of (95 °C 1 min, 58 °C 1 min, 72 °C 1.5 min)<br>• 72 °C 5 min |
| | EtMIC2NR | CCTTCAGGATGACTGTTGAGTGTC | | |
| *etsag1* | EtSAG1F | ATGGCTCGTCTTTCTTTTGTTTCTC | 1101 | • 95 °C 5 min<br>• 30 cycles of (95 °C 1 min, 55 °C 1 min, 72 °C 1.5 min)<br>• 72 °C 5 min |
| | EtSAG1R | ATCTCCGCTTTCGCTCTCTTTTAG | | |
| | EtSAG1NF | CAGGATTACCCAACAGCAGGTG | 979 | • 95 °C 5 min<br>• 35 cycles of (95 °C 1 min, 58 °C 1 min, 72 °C 1.5 min)<br>• 72 °C 5 min |
| | EtSAG1NR | GGACGACTGGAGAAACTCCGC | | |
