# Supplementary material for: Genetic Diversity of Microneme Protein 2 and Surface Antigen 1 of Eimeria tenella
Source: Genes (Basel). 2021 Sep 15;12(9):1418. doi: 10.3390/genes12091418 (PMC8470435; doi:10.3390/genes12091418)
Supplement: Supplementary file 1 [file genes-12-01418-s001.zip › Eimeria_Vo et al._Supplementary Materials_Table S2.pdf]

Table S2. List of sequences analyzed in this study.

Gene	Origin	Number of sequences	GenBank accession numbers
etmic2	Houghton	1	XM_013377912.1
	China	21	KY117173.1–KY117192.1, AF111839.1
	India	1	FJ807654.1
etsag1	Houghton	1	AJ586531.2
	China	21	KY117193.1–KY117212.1, DQ327836.1
	India	2	KF718807.1, KF718808.1
